# Reactive Metabolites and AGE-RAGE-Mediated Inflammation in Patients following Liver Transplantation

**DOI:** 10.1155/2013/501430

**Published:** 2013-05-22

**Authors:** Thorsten Brenner, Thomas H. Fleming, David Spranz, Peter Schemmer, Thomas Bruckner, Florian Uhle, Eike O. Martin, Markus A. Weigand, Stefan Hofer

**Affiliations:** ^1^Department of Anesthesiology, University of Heidelberg, Im Neuenheimer Feld 110, 69120 Heidelberg, Germany; ^2^Department of Medicine I and Clinical Chemistry, University of Heidelberg, Im Neuenheimer Feld 410, 69120 Heidelberg, Germany; ^3^Department of General and Transplant Surgery, University of Heidelberg, Im Neuenheimer Feld 110, 69120 Heidelberg, Germany; ^4^Institute of Medical Biometry and Informatics, University of Heidelberg, Im Neuenheimer Feld 305, 69120 Heidelberg, Germany; ^5^Department of Anesthesiology and Intensive Care Medicine, University of Gießen, Rudolf-Buchheim-Straße 7, 35392 Gießen, Germany

## Abstract

Recent investigations have indicated that reactive metabolites and AGE-RAGE-mediated inflammation might play an important role in the pathogenesis of ischemia-reperfusion injury in liver transplantation. In this observational clinical study, 150 patients were enrolled following liver transplantation from deceased donors. The occurrence of short-term complications within 10 days of transplantation was documented. Blood samples were collected prior to transplantation, immediately after transplantation, and at consecutive time points, for a total of seven days after transplantation. Plasma levels of methylglyoxal were determined using HPLC, whereas plasma levels of L-arginine, asymmetric dimethylarginine, advanced glycation endproducts-carboxylmethyllysine, soluble receptor for advanced glycation endproducts, and total antioxidant capacity were measured by ELISA. Patients following liver transplantation were shown to suffer from increased RAGE-associated inflammation with an AGE load mainly dependent upon reactive carbonyl species-derived AGEs. In contrast, carboxylmethyllysine-derived AGEs were of a minor importance. As assessed by the ratio of L-arginine/asymmetric dimethylarginine, the bioavailability of nitric oxide was shown to be reduced in hepatic IRI, especially in those patients suffering from perfusion disorders following liver transplantation. For the early identification of patients at high risk of perfusion disorders, the implementation of asymmetric dimethylarginine measurements in routine diagnostics following liver transplantation from deceased donors should be taken into consideration.

## 1. Introduction

Liver transplantation (LTPL) is a routinely used therapeutic option in patients with end-stage liver disease. Due to improvements in medical care in the last decade, the long-term outcome of patients following LTPL has improved. Nevertheless, failure or impaired function of the liver graft has been observed. As the graft can suffer from acute injury as a consequence of vascular clamping and declamping procedures during transplantation, ischemia-reperfusion injury (IRI) has been proposed as a risk factor for posttransplantation organ recovery [1–6]. Several investigations have suggested that reactive oxygen species (ROS: e.g., O_2_^−^) may be important mediators of reperfusion injury in IRI [7–12], leading to protein and deoxyribonucleic acid (DNA) oxidation, lipid peroxidation, and the interaction with other intracellular and extracellular radical species, such as reactive nitrogen species (RNS: e.g., nitric oxide/NO) [13–15]. This interaction is known to produce the potent cytotoxic oxidizing and nitrating species peroxynitrite (ONOO^−^) and its conjugate peroxynitrous acid (HONOO). However, NO has also been demonstrated to be an important hepatoprotective molecule, as decreased NO bioavailability is associated with impaired microcirculatory blood flow and increased mortality in mouse IRI models [16, 17]. NO is synthesized from the amino acid L-arginine (L-arg) by the action of nitric oxide synthase (NOS), the activity of which can be influenced by endogenous NOS inhibitors such as asymmetric dimethylarginine (ADMA) [18], for which the liver has been described as an important organ in metabolism [19].

Beside RNS, the amount of circulating ROS is closely related to the generation of reactive carbonyl species (RCS: e.g., methylglyoxal/MG), since the formation as well as the breakdown of RCS is associated with an increased free radical generation [20–22]. Reactive carbonyl species (RCS) are a very heterogeneous group of reactive low molecular weight carbonyls, which are able to interact with various biomolecules, such as proteins, DNA, or phospholipids, resulting in structural distortions and functional impairment [23]. The detrimental effects of RCS are therefore comparable to those caused by ROS accumulation. Accordingly, plasma levels of RCS were suggested to be reliable markers to predict the level of IRI [24]. MG belongs to a class of reactive carbonyl species (RCS) known as *α*-oxoaldehydes [25]. These RCS contain two adjacent carbonyl groups (which are therefore named dicarbonyls) making them a highly reactive class of RCS [26]. MG formation primarily results from a spontaneous degradation of triosephosphates (glyceraldehyde-3-phosphate (GAD3P), dihydroxyacetone phosphate (DHAP)). Therefore, an increased formation of alpha-dicarbonyls was anticipated in cases of an increased glycolytic flux or enhanced dependence on glycolysis for energy. Moreover, an increased formation of ROS (e.g., O_2_^−^) is able to inhibit GAD3P-dehydrogenase (GAD3PDH) activity throughout different pathways, resulting in an accumulation of upstream intermediates and the subsequent increased formation of MG [27, 28].

**Figure 1:**
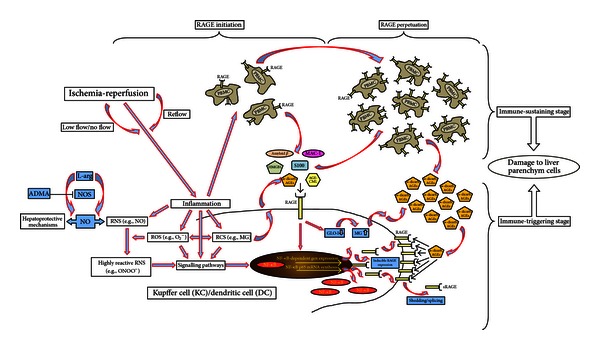
Proposed cellular activation via the AGE/RAGE pathway in hepatic ischemia-reperfusion injury (IRI).

Through the posttranslational modification of proteins, to form advanced glycation endproducts (AGEs), RCS can mediate inflammation through the receptor for advanced glycation endproducts (RAGE) [25]. AGE-modified proteins represent a potpourri of very heterogeneous chemical structures which are generated throughout multistage cross-linked pathways, leading to different subgroups of AGEs (e.g., AGE-N*ε*(carboxymethyl)lysine (CML), alpha-dicarbonyl-derived AGEs) [29, 30]. Glucose and other reducing sugars are important glycating agents, whereas alpha-dicarbonyls, in particular methylglyoxal (MG), seem to be the most relevant [31]. The critical role of MG, as well as the resulting alpha-dicarbonyl-derived AGE formation, is well described for chronic inflammatory diseases (e.g., diabetes mellitus). It has been shown that diabetes-associated complications such as nephropathy, neuropathy and, retinopathy are associated with the cellular AGE load [32–35]. However, sophisticated analyses of AGE formation as well as resulting AGE/RAGE interactions in patients following LTPL have yet to be investigated. The proposed cellular activation pathways in hepatic IRI are presented in Figure 1.

The aim of this study was to investigate whether dysfunction of the L-arg/NO-pathway, generation of ROS and RCS, and the resulting AGE-RAGE-mediated inflammation might play an important role in the pathogenesis of IRI in LTPL. The prognostic values of each of the different parameters in patients following LTPL in the initial phase after transplantation would be evaluated as means of identifying liver graft dysfunction.

## 2. Materials and Methods

The observational clinical study was approved by the local ethics committee (Ethics Committee of the Medical Faculty of Heidelberg; Trial-Code No.: S055-2009/German, Clinical Trials Register ID: DRKS00003434). Study patients signed written informed consent. In total, 150 patients following LTPL from deceased donors were enrolled from May 2009 until May 2011. The management of LTPL patients was performed according to Heidelberg Manual for Liver Transplantation [37]. Relevant baseline data, clinical data, and routine blood parameters were collected. Patients were reevaluated for short-term complications for 10 days following LTPL as described earlier [38]. Patients who did not develop any complications within the 10-day observation period served as a control group. Blood samples from LTPL patients were collected prior to transplantation (Pre) and immediately after the surgical procedure (T0), as well as 1 day (T1), 3 days (T3), 5 days (T5), and 7 days (T7) later. Plasma of all study participants was immediately obtained by centrifugation, transferred into cryotubes, and stored at −80°C until further processing. Plasma concentrations of L-arg, ADMA, AGE-CML, sRAGE, and TAC were performed using ELISA kits according to the manufacturer's instructions (ADMA & L-arg: Immundiagnostik, Bensheim, Germany; AGE-CML: MicroCoat, Bernried, Germany; sRAGE: R&D Systems, Minneapolis, MN, USA; TAC: Biocat, Heidelberg, Germany). For MG measurements, 1 ml of plasma was added to a cryovial containing 100 *µ*l of trichloroacetic acid, snap frozen in liquid nitrogen, and stored at −80°C until further processing. The concentration of MG was determined by derivatization with 1,2-diamino-4,5-dimethoxybenzene and HPLC of the quinoxaline adduct by fluorescence detection [39, 40].

The resulting study data were entered into an electronic database (Microsoft Excel 2010, Microsoft Corporation, Redmond, WA, USA) and evaluated using SPSS software (Version 20.0, SPSS Inc., Chicago, IL, USA). Categorical data were summarized by means of absolute and relative frequencies. Quantitative data were summarized using the median with quartiles. The Kolmogorov-Smirnov test was applied to check for normal distribution. Due to nonnormally distributed data, nonparametric methods for evaluation were used (chi-squared test for categorical data, Mann-Whitney test for continuous data). Furthermore, a receiver operating characteristic curve was established with suitable parameters, in order to create cut-off values to determine the prognostic value of each parameter with regard to the development of complications following LTPL. Comparisons of the areas under two or more correlated ROC curves were performed, as described by DeLong et al. [41]. Correlation analysis was performed by calculating Pearson's correlation coefficient (*ρ*). A *P* value < 0.05 was considered statistically significant. Concerning symbolism and higher orders of significance: *P < 0.05, **P < 0.01, and ***P < 0.001.

## 3. Results

### 3.1. Baseline Data

Baseline data of the 150 patients undergoing LTPL from deceased donors are presented in detail in Table 1.

### 3.2. Oxidative Stress in Hepatic IRI

As assessed by TAC plasma levels, patients with end-stage liver disease suffer from increased oxidative stress prior to transplantation. Following the transplantation procedure, TAC plasma levels further increased, showing peak levels 24 h after transplantation. TAC plasma levels were observed to decline until T7 without decreasing below initial values (Table 2, Figure S1 available online at http://dx.doi.org/10.1155/2013/501430). 

### 3.3. RCS and AGE/RAGE-Mediated Inflammation in Hepatic IRI

**Figure 2:**
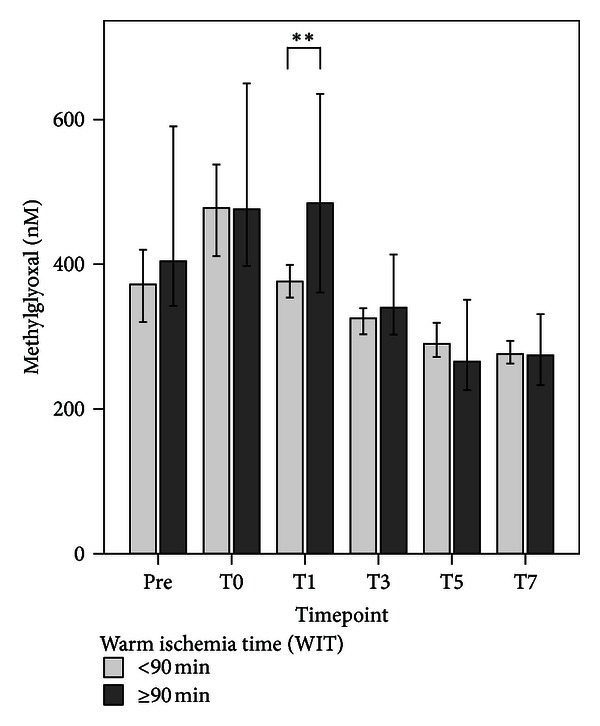
Comparisons of methylglyoxal (MG) measurements in patients following liver transplantation (LTPL) from deceased donors with a warm ischemia time less (*n* = 114; light grey bar) or more (*n* = 36; dark grey bar) than 90 minutes at six different timepoints: prior to transplantation (Pre) and immediately after the end of the surgical procedure (T0), as well as 1 day (T1), 3 days (T3), 5 days (T5), and 7 days (T7) later. Data in bar charts are given as medians and the 95% CI. Concerning symbolism and higher orders of significance: **P < 0.01.

As assessed by sRAGE plasma levels, patients with end-stage liver disease were shown to suffer from increased RAGE-associated inflammation prior to transplantation. sRAGE plasma levels further increased following the transplantation procedure, reaching peak levels at T0 with continuously declining levels until T7 (Table 2, Figure S2a). Analogously, plasma levels of CML-derived AGEs (Table 2, Figure S2b) as well as MG (Table 2, Figure S2c) were increased prior to transplantation. Following the transplantation procedure, leveling of CML-derived AGEs and MG differed substantially: CML-derived AGEs decreased initially and returned to baseline levels until T7. In contrast, MG increased in the early posttransplantation period and reached its peak level at T0. Afterwards, MG plasma levels declined continuously and decreased below baseline levels. Therefore, cellular AGE load in hepatic IRI seems to be mainly dependent on RCS-derived AGEs, whereas CML-derived AGEs were shown to be of minor importance. Moreover, the amount of RCS-derived AGEs was shown to be influenced by the duration of warm ischemia time (WIT), since LTPL patients with a WIT ≥ 90 min revealed significantly increased plasma levels of MG at T1 in comparison to LTPL patients with a WIT < 90 min (Figure 2).

### 3.4. NO Homeostasis in Hepatic IRI

**Figure 3:**
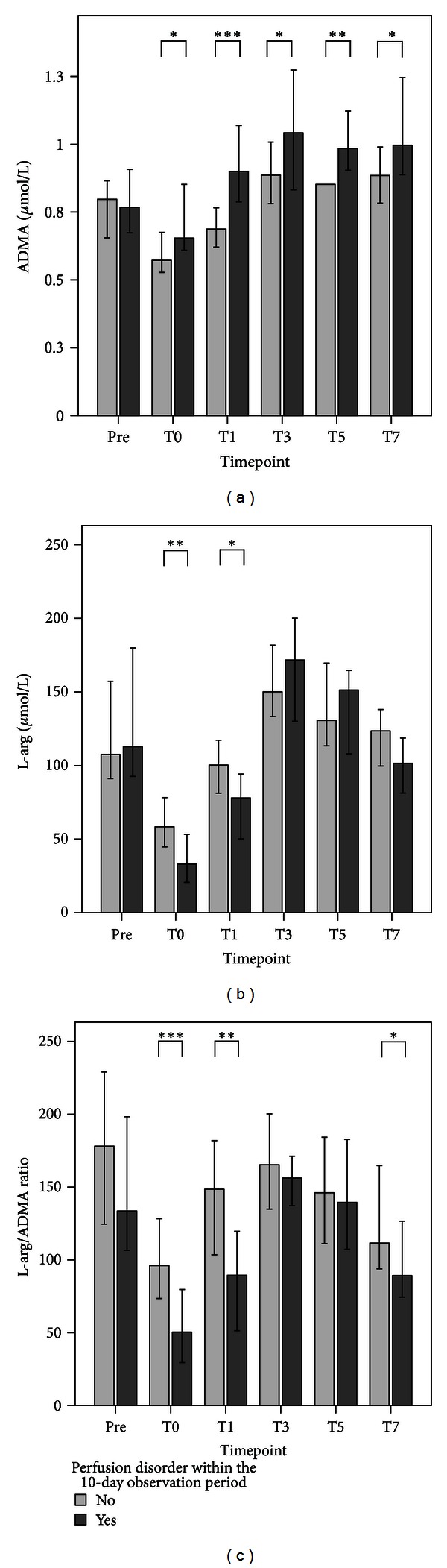
Plasma levels of (a) asymmetric dimethylarginine (ADMA), (b) L-arginine (L-arg), and (c) the ratio of both (L-arg/ADMA) in patients who suffered from a perfusion disorder (*n* = 37; dark grey bar) in comparison to those who did not develop any complications (*n* = 52; light grey bar) within the 10-day observation period following liver transplantation (LTPL) from deceased donors at six different timepoints: prior to transplantation (Pre) and immediately after the end of the surgical procedure (T0), as well as 1 day (T1), 3 days (T3), 5 days (T5), and 7 days (T7) later. Data in bar charts are given as medians and the 95% CI. Concerning symbolism and higher orders of significance: *P < 0.05; **P < 0.01; and ***P < 0.001.

**Figure 4:**
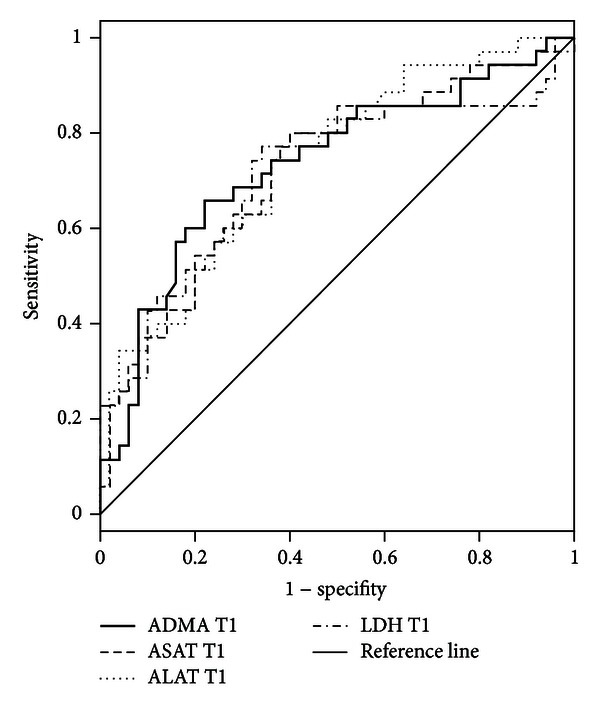
Receiver operating characteristic (ROC) curves for asymmetric dimethylarginine (ADMA), aspartate aminotransferase (ASAT), alanine aminotransferase (ALAT), and lactate dehydrogenase (LDH) measurements 1 day after the end of the surgical procedure (T1) in plasma samples of patients who suffered from a perfusion disorder (*n* = 37) in comparison to those who did not develop any complication (*n* = 52) within the 10-day observation period following liver transplantation (LTPL) form deceased donors.

Plasma levels of ADMA were shown to be increased already prior to transplantation with temporarily decreasing levels in the early phase after transplantation. Later, ADMA plasma levels exceeded baseline levels and reached a steady state at T3 (Table 2, Figure S3a). Plasma levels of L-arg showed a comparable leveling (Table 2, Figure S3b). Accordingly, plasma levels of L-arg and ADMA revealed a moderate positive correlation (*ρ* = 0.381). The resulting ratio of both parameters (L-arg/ADMA ratio) revealed a reduced NO bioavailability in patients undergoing LTPL before as well as after the transplantation procedure, whereas it was most pronounced immediately after the end of the transplantation procedure (Table 2, Figure S3c). Further subgroup analysis revealed that NO bioavailability was further reduced in patients with a perfusion disorder in comparison to patients with no complications (Figures 3(a)–3(c)). It could be demonstrated that ADMA at T1 (ROC-AUC: 0.73; Cutoff: 0.82 *µ*mol/l → Sensitivity 0.66; 1-Specifity 0.22) is able to differentiate between patients with a perfusion disorder (*n* = 37; 24.7% → consisting of, for example, thrombosis/stenosis of hepatic artery (*n* = 11; 7.3%) or portal vein (*n* = 2; 1.3%), major bleeding with the need for reoperation (*n* = 18; 12.0%)) and patients with no complications (*n* = 52; 34.7%), compared to either aspartate aminotransferase (ASAT) at T1 (ROC area under the curve (AUC): 0.73; Cut-Off: 934 U/l → Sensitivity 0.80; 1-Specifity 0.40), alanine aminotransferase (ALAT) at T1 (ROC-AUC: 0.74; Cut-Off: 578 U/l → Sensitivity 0.77; 1-Specifity 0.38), or lactate dehydrogenase (LDH) at T1 (ROC-AUC: 0.71; Cut-Off: 656 U/l; Sens. 0.77; 1-Specifity 0.34), as assessed by area AUC comparisons of the related ROC curves (contrast test results: *P* = 0.97) (Figure 4).

## 4. Discussion

The pathophysiology of hepatic IRI is described to be a biphasic process [6] involving ischemia, in which the lack of any oxygen supply and depletion of adenosine trisphosphate (ATP) induces hepatocellular damage, and the reperfusion injury, which contributes to abundant immunoinflammatory responses; these likewise result in hepatocellular cell death [3, 4, 42–45]. IRI-associated immunoinflammatory processes consist of two related stages: (i)* immune-triggering stage:* cellular stress or damage due to hepatic IRI leads to the generation of alarmins (damage-associated molecular patterns/DAMP); these danger molecules are known to activate liver resident Kupffer cells (KC) as well as dendritic cells (DC) throughout the corresponding pattern recognition receptors (PRR: e.g., RAGE), subsequently boosting immunoinflammatory responses, and (ii)* immune-sustaining stage:* circulating mononuclear and polymorphonuclear cells become activated and recruited into sites of IRI in order to sustain local immune responses, resulting in an amplification of local tissue damage (Figure 1) [45].

Oxidative stress is described to be a key mechanism in the pathophysiology of hepatic IRI in liver surgery and liver transplantation, contributing to the overall organ damage in various degrees [46]. Moreover, oxidative stress plays a crucial role in the introduction and progression of various liver diseases [47]. Accordingly, patients with end-stage liver diseases within the present investigation showed increased levels of TAC already prior to transplantation. 

ROS are considered to be major causes of oxidative stress-associated epithelial injury [45]. Alternative mediators to ROS are RCS, a heterogeneous group of small molecular weight carbonyls. These compounds are formed endogenously, and their production is closely linked to the formation of ROS [20–22]. The harmful effects of RCS are similar to the detrimental effects caused by ROS accumulation and have in the past been either mistaken or overlooked. Compared to ROS, RCS are stable and diffuse within or even escape from the site of origin and can attack targets far from their site of formation [23]. The critical role of RCS in patients following LTPL has not been fully investigated. Waller et al. have shown that the plasma levels of carbonyls in an isolated organ perfusion of transplanted kidney were a reliable marker to predict the level of IRI [24]. This finding is supported by the data in this study, in which it was shown that the plasma MG levels peaked at T0 followed by a continuous decline until T7, which was in accordance with the expected progression of the reperfusion injury. Moreover, MG was shown to be increased depending on the duration of WIT.

The harmful effects of dicarbonyls, such as MG, are substantially mediated through their ability to form AGE-modified proteins [48, 49]. AGE proteins are known to be potent ligands of RAGE [25, 50–52] and result from the nonenzymatic reaction of the dicarbonyl with the free amino groups of the N-terminal residue of a protein, as well as the lysyl side chain and guanidine groups of arginine residues. The glycation of proteins in this manner involves a complex series of parallel and sequential reactions referred to as the Maillard reaction. Since MG is characterized by an extremely high AGE-generating potency [25, 30], measurements of MG as a precursor of dicarbonyl-derived AGE seem to be eligible for the indirect assessment of the dicarbonyl-derived AGE load.

In patients with end-stage liver diseases, plasma levels of AGE-CML were shown to be related positively to disease severity and negatively to residual liver function, which is consistent with the liver representing a major site of AGE-CML catabolism [53–55]. It was shown in this study that plasma levels of AGE-CML were increased prior to the transplantation procedure; however, they temporarily decreased in the early phase after transplantation and then returned to baseline, suggesting that AGE-CML seems not to be a key mediator in hepatic IRI. In contrast, dicarbonyl-derived AGE formation was shown to be of major importance in the pre- as well as early posttransplantation period. This might be due to the fact that both settings (liver diseases, hepatic IRI) are known to be characterized by a prooxidative environment due to ROS production [46, 47].

RAGE is a member of the PRR family and is localized both on liver resident KC/DC and circulating mononuclear and polymorphonuclear cells. Following activation by its ligands (e.g., high mobility group box protein-1 (HMGB-1), amyloid *β*, AGEs, and S100 proteins/*RAGE initiation*), RAGE is able to perpetuate nuclear factor kappa-B (NF*κ*B)-p65-activation [50, 52] and leads to oxidative stress, as well as the propagation of inflammatory responses [50, 56] (*RAGE perpetuation*) (Figure 1). 

Within this study, measurements of sRAGE were performed in order to assess the degree of RAGE-mediated inflammation [57]. Patients with the need for liver transplantation were shown to suffer from increased RAGE activation, which increased further in the early phase following LTPL. Therefore, RAGE-mediated inflammation seems to be of relevance in patients with end-stage liver disease and is further increased due to the transplantation procedure. Furthermore, the leveling of plasma sRAGE, TAC, and MG was shown to be comparable. This may support our hypothesis (Figure 1) that these different aspects are connected in the transplantation setting. Concerning the critical role of sRAGE itself, we cannot at present provide any additional information, as there is a considerable and controversial debate as to whether sRAGE might function as a decoy by preventing ligands from interacting with cellular RAGE [58–61] or forms potent proinflammatory complexes with other mediators of inflammation (e.g., the *β*-integrin MAC-1) resulting in sustained cell activation [62].

Beside ROS and RCS, RNS represent a further group of reactive metabolites. As described earlier, ROS and RNS are both part of an oxidative-inflammatory vicious cycle, as O_2_^−^ in combination with NO is able to produce the potent cytotoxic species peroxynitrite (ONOO^−^). Peroxynitrite is known to induce severe tissue damage in ongoing shock, inflammation, or IRI [63]. Moreover, NO has been shown to stimulate mitochondrial production of H_2_O_2_ and O_2_^o−^ due to the inhibition of cytochrome c oxidase [64]. In turn, H_2_O_2_ leads to an upregulation of inducible NOS (iNOS) as a result of NF*κ*B induction [65]. However, conversely, NO has also been shown to be an important hepatoprotective molecule in the postischemic liver, since endothelial NOS^−/−^ (eNOS)- or N(omega)-nitro-L-arginine-methyl ester (L-NAME)-treated mice are more sensitive to the damaging effects of IRI [16, 17]. This protective effect was assigned to a perfusion-related function, since NO is able to maintain sinusoidal perfusion in the postischemic liver due to counteracting the effects of other vasoconstrictor substances, such as phenylephrine or endothelin. Therefore, adequate plasma levels of NO are recommended, especially in cases of IRI, in order to avoid O_2_^−^-mediated NO removal/consumption. The bioavailability of NO depends on two major factors: (i)* L-arg:* NO is synthesized from the amino acid L-arg by the action of NOS, resulting in a proportional bioavailability of L-arg and NO [18], and (ii)* ADMA:* activity of NOS can be influenced by endogenous inhibitors such as ADMA, resulting in an inversely proportional bioavailability of ADMA and NO [66]. In terms of the “L-arginine-paradox”, plasma levels of L-arg and ADMA are described to be subject to closely intertwined regulatory processes [67, 68]. The “L-arginine-paradox” describes an ADMA-induced right shift of the NOS concentration-response curve for L-arg, so that only small changes in L-arg plasma concentrations may cause marked changes in NOS activity in order to avoid a relevant lack of NO [67]. Accordingly, LTPL patients within this investigation showed a comparable leveling of both parameters, resulting in a moderate positive correlation according to Pearson's correlation analysis. Since ADMA seems to be a driving force in the regulatory processes determining NO bioavailability, one should have a long close look at its metabolism.

ADMA is primarily metabolized to citrulline and dimethylamine by the enzyme dimethylaminohydrolase (DDAH), which has high activity in the liver and kidneys [69–71]. Moreover, ADMA competes with L-arg, symmetrical dimethylarginine (SDMA), and L-lysine for the cationic amino acid transporters (CAT)-dependent transport across the cell membrane, especially in hepatocytes [72]. Elevated concentrations of ADMA have been found in patients suffering from hepatic dysfunction, as well as end-stage renal disease, and the liver has been postulated to play an important role in the absorption and degradation of ADMA [19, 73]. Previously, we have shown increased plasma levels of ADMA in patients with a sepsis-associated acute liver failure. Furthermore, ADMA was shown to be of outcome-relevance, since plasma levels of ADMA served as early predictors for survival in these patients [36]. With respect to liver surgery in general, ADMA plasma levels have been reported to be elevated in patients following major liver surgery due to a temporary hepatic insufficiency with a subsequently reduced ADMA clearance [74]. Accordingly, patients with end-stage liver diseases and the need for liver transplantation were described to suffer from elevated plasma levels of ADMA prior to transplantation [75, 76]. Moreover, in patients following liver transplantation, increased ADMA concentrations were theorized to be a potential marker of dysfunctions in the liver graft [75, 76]. Both observations can be supported by our study, since plasma levels of ADMA in patients undergoing LTPL were elevated at pretransplantation compared to healthy volunteers. Moreover, ADMA plasma levels were shown to be significantly elevated in patients suffering from a perfusion disorder in comparison to those patients without any complications.

## 5. Conclusions

It was observed that AGE-RAGE-mediated inflammation is of relevance in the pathophysiology of hepatic IRI. In this setting cellular AGE load was demonstrated to be mainly dependent on RCS-derived AGEs, whereas CML-derived AGEs seem to be of minor importance. Moreover, NO bioavailability was shown to be reduced in hepatic IRI, especially in those patients suffering from a perfusion disorder following orthotopic liver transplantation. The endogenous NOS inhibitor ADMA was therefore identified as an early predictor for a perfusion disorder in patients following orthotopic liver transplantation from deceased donors. The implementation of ADMA measurements in routine diagnostics following liver transplantation from deceased donors should therefore be taken into consideration.

## Supplementary Material

Figure S1. Plasma levels of total antioxidant capacity (TAC) in 150 patients following liver transplantation (LTPL) from deceased donors prior to transplantation (Pre), immediately after the end of the surgical procedure (T0), as well as 1 day (T1), 3 days (T3), 5 days (T5) and 7 days (T7) later. Data in bar charts are given as medians and the 95% CI. Median plasma level of TAC in healthy volunteers (n=30; red striped line) is presented (previously unpublished data of RAMMSES-Trial / German Clinical Trials Register: DRKS00000505).
Figure S2. Plasma levels of (a) soluble receptor for advanced glycation endproducts (sRAGE), (b) advanced glycation endproducts-carboxymethyllysine (AGE-CML) and (c) methylglyoxal (MG) in 150 patients following liver transplantation (LTPL) from deceased donors prior to transplantation (Pre), immediately after the end of the surgical procedure (T0), as well as 1 day (T1), 3 days (T3), 5 days (T5) and 7 days (T7) later. Data in bar charts are given as medians and the 95% CI. Median plasma levels of sRAGE, AGE-CML as well as MG in healthy volunteers (n=30; red striped line) are presented (previously unpublished data of RAMMSES-Trial / German Clinical Trials Register: DRKS00000505).
Figure S3. Plasma levels of (a) asymmetric dimethylarginine (ADMA), (b) L-arginine (L-arg) and (c) the ratio of both (L-arg/ADMA) in 150 patients following liver transplantation (LTPL) from deceased donors prior to transplantation (Pre), immediately after the end of the surgical procedure (T0), as well as 1 day (T1), 3 days (T3), 5 days (T5) and 7 days (T7) later. Data in bar charts are given as medians and the 95% CI. As already published throughout our workgroup, median plasma levels of the three parameters in healthy volunteers (n=30; red striped line) are presented [36].

## Figures and Tables

**Table 1:** Baseline data of 150 patients undergoing liver transplantation (LTPL) from deceased donors.

Baseline data	
Urgency	

Nonurgent	123 (82.0%)
High urgency (HU)	27 (18.0%)

Surgical specialties	

First-time LTPL	121 (80.7%)
Re-LTPL	29 (19.3%)

Disease severity	

Lab MELD score	21.5 (11.0–34.0)

Ischemia times^a^	

Cold ischemia time (CIT) (min)	685 (596–722)
Warm ischemia time (WIT) (min)	62 (50–80)

Primary liver diseases in patients undergoing first-time LTPL	
(*n* = 121)	

Ethyl-toxic cirrhosis	20 (16.5%)
Viral hepatitis	13 (10.7%)
Hepatocellular carcinoma (HCC)	36 (29.8%) *Origin*: viral hepatitis 18 (14.9%), ethyl-toxic cirrhosis 16 (13.2%), cryptogenic 2 (1.7%)
Others	52 (42.1%)

Data are presented as number (%) or as median with quartiles (Q1–Q3).

^a^Definitions: the CIT was defined as the period between donor aortic cross-clamping during organ procurement and graft removal from iced water at the recipient site.

The allograft rewarming time between graft removal from iced water at the recipient site and portal reperfusion was regarded as the WIT.

**Table 2:** Plasma levels of total antioxidant capacity (TAC), soluble receptor for advanced glycation endproducts (sRAGE), advanced glycation endproducts-carboxymethyllysine (AGE-CML), methylglyoxal (MG), asymmetric dimethylarginine (ADMA), L-arginine (L-arg), and the ratio of both (L-arg/ADMA) in 150 patients following liver transplantation (LTPL) form deceased donors.

Timepoints		Pre	T0	T1	T3	T5	T7	Healthy controls^a^
TAC	(mM)	**0.062**; 0.018–0.106	**0.093**; 0.052–0.171	**0.105**; 0.058–0.229	**0.104**; 0.050–0.189	**0.087**; 0.049–0.188	**0.079**; 0.040–0.180	**0.017**; 0.004–0.030
sRAGE	(pg/mL)	**1447.2**; 944.6–2507.7	**1982.3**; 1271.9–3253.4	**1708.0**; 1017.4–3050.1	**1199.3**; 686.1–2391.0	**900.1**; 563.8–1782.7	**802.6**; 436.1–1503.3	**430.0**; 276.6–625.8
AGE-CML	(ng/mL)	**1810.3**; 1144.0–3327.7	**1164.9**; 890.9–1558.5	**1430.6**; 1123.4–1963.6	**1692.0**; 1310.6–2362.7	**1823.3**; 1376.2–2470.8	**1817.5**; 1310.3–2650.3	**1249.3**; 1154.5–1385.9
MG	(nM)	**373.1**; 291.3–521.7	**477.9**; 339.3–627.3	**388.9**; 318.2–554.5	**328.4**; 271.1–390.7	**289.4**; 229.4–356.1	**275.5**; 233.1–329.2	**105.8**; 98.7–131.7
ADMA	(*µ*mol/L)	**0.77**; 0.61–0.93	**0.62**; 0.51–0.77	**0.76**; 0.63–0.94	**0.90**; 0.74–1.16	**0.91**; 0.75–1.10	**0.90**; 0.76–1.13	**0.43**; 0.37–0.51
L-arg	(*µ*mol/L)	**138.3**; 87.3–199.3	**54.1**; 30.7–85.2	**99.1**; 66.4–141.6	**164.8**; 123.9–202.5	**143.9**; 108.4–189.6	**119.2**; 84.1–163.6	**112.6**; 100.8–133.0
L-arg/ADMA	(none)	**177.0**; 113.2–276.5	**81.5**; 48.3–134.6	**125.0**; 82.6–183.9	**163.9**; 126.4–230.4	**148.5**; 109.2–208.4	**117.8**; 88.3–172.5	**273.5**; 218.8–329.3

Data are presented as median with quartiles (Q1–Q3).

^a^As already published throughout our workgroup, median plasma levels of ADMA, L-arg, and the ratio of both (L-arg/ADMA) in healthy volunteers are presented [36]. Plasma levels of TAC, sRAGE, AGE-CML, and MG in healthy controls are also presented (previously unpublished data of RAMMSES-Trial/German Clinical Trials Register: DRKS00000505).
