# Supplementary figures and images for: Reactive Metabolites and AGE-RAGE-Mediated Inflammation in Patients following Liver Transplantation
Source: Mediators Inflamm. 2013 May 22;2013:501430. doi: 10.1155/2013/501430 (PMC3677670; doi:10.1155/2013/501430)

**Supplemental Figures.**

**Figure S1.**

**
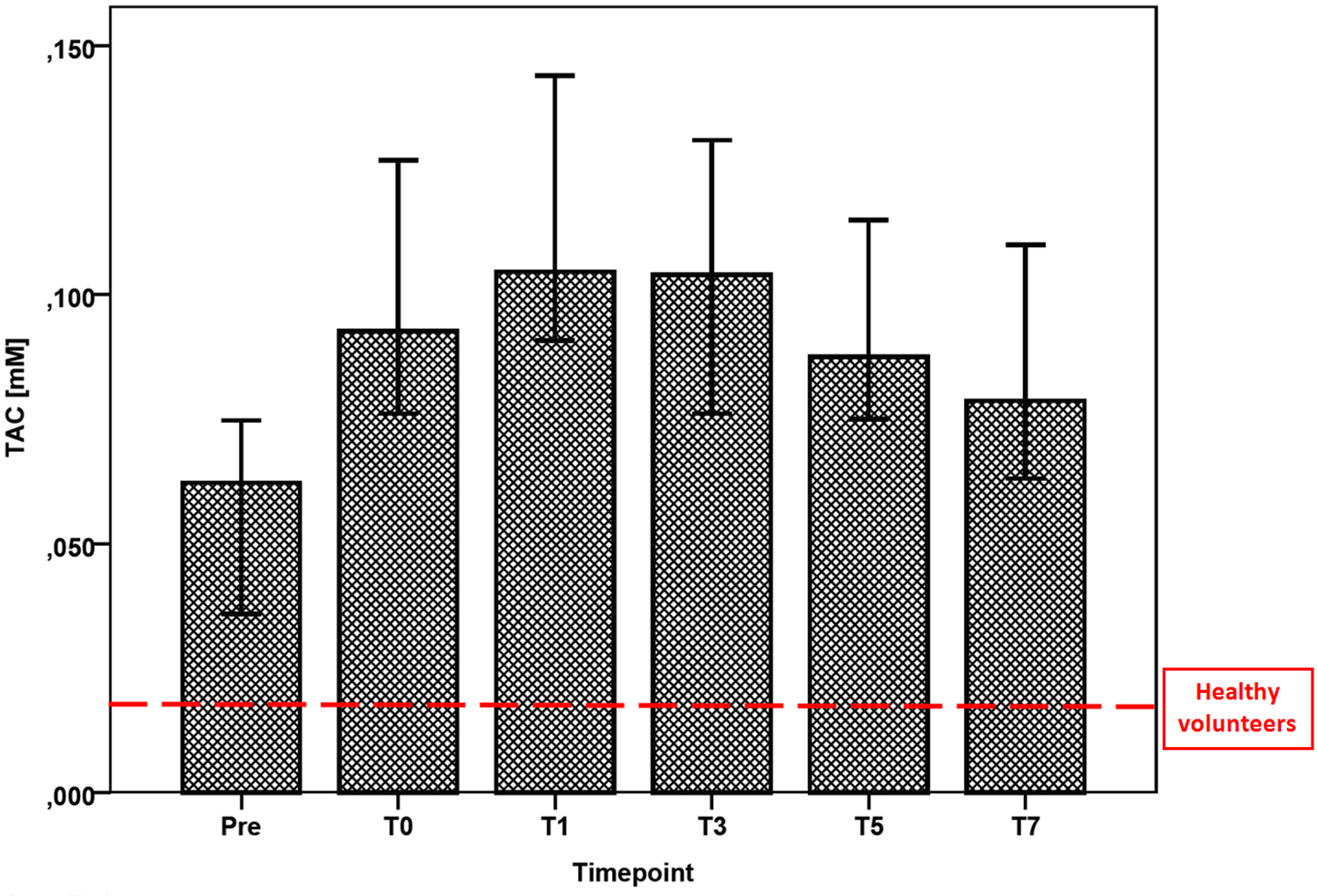
**

**Figure S2.**


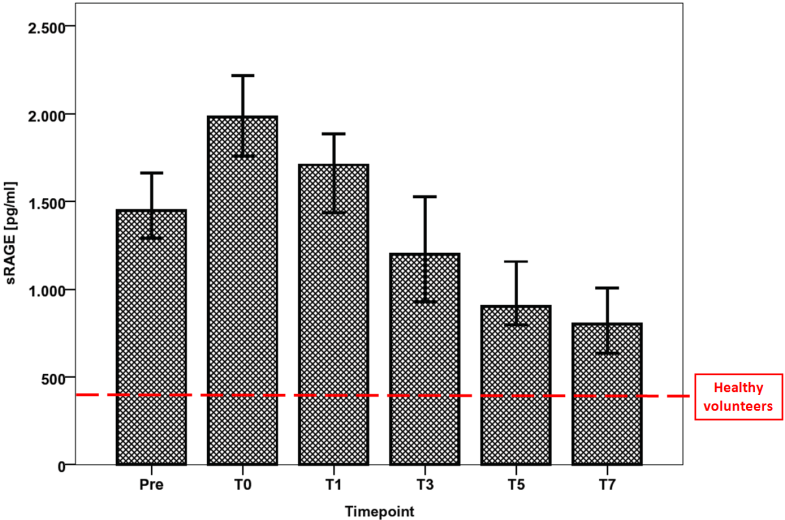
**(a.)**


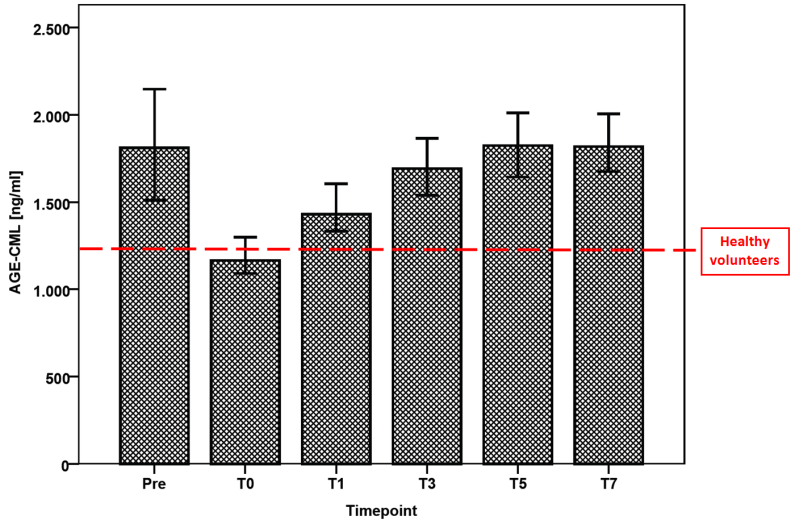
**(b.)**


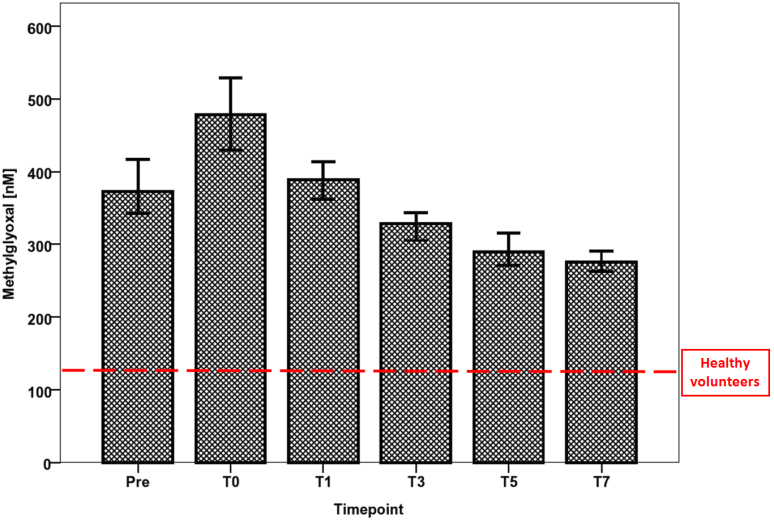
**(c.)**

**Figure S3.**


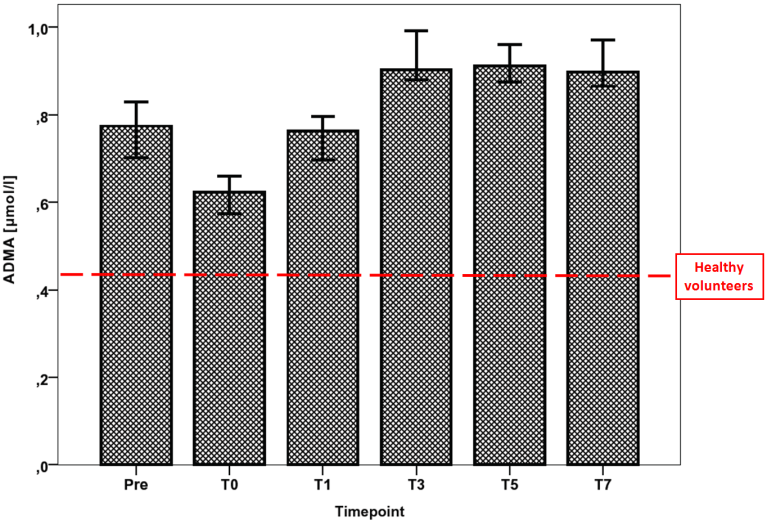
**(a.)**


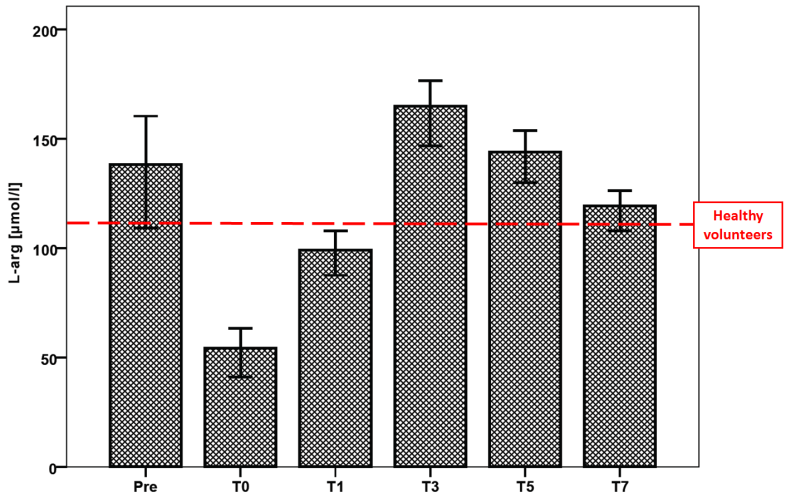
**(b.)**

**(c.)**

**
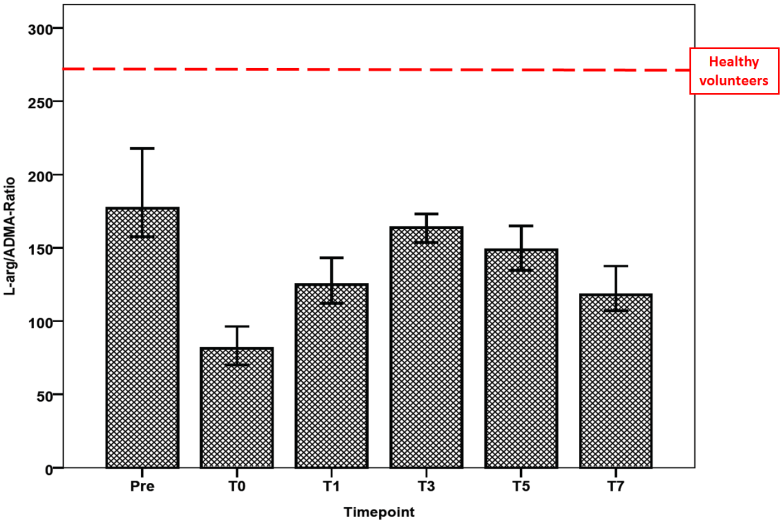
**

Supplement: Supplementary file 1 — Figure S1. Plasma levels of total antioxidant capacity (TAC) in 150 patients following liver transplantation (LTPL) from deceased donors prior to transplantation (Pre), immediately after the end of the surgical procedure (T0), as well as 1 day (T1), 3 days (T3), 5 days (T5) and 7 days (T7) later. Data in bar charts are given as medians and the 95% CI. Median plasma level of TAC in healthy volunteers (n=30; red striped line) is presented (previously unpublished data of RAMMSES-Trial / German Clinical Trials Register: DRKS00000505). Figure S2. Plasma levels of (a) soluble receptor for advanced glycation endproducts (sRAGE), (b) advanced glycation endproducts-carboxymethyllysine (AGE-CML) and (c) methylglyoxal (MG) in 150 patients following liver transplantation (LTPL) from deceased donors prior to transplantation (Pre), immediately after the end of the surgical procedure (T0), as well as 1 day (T1), 3 days (T3), 5 days (T5) and 7 days (T7) later. Data in bar charts are given as medians and the 95% CI. Median plasma levels of sRAGE, AGE-CML as well as MG in healthy volunteers (n=30; red striped line) are presented (previously unpublished data of RAMMSES-Trial / German Clinical Trials Register: DRKS00000505). Figure S3. Plasma levels of (a) asymmetric dimethylarginine (ADMA), (b) L-arginine (L-arg) and (c) the ratio of both (L-arg/ADMA) in 150 patients following liver transplantation (LTPL) from deceased donors prior to transplantation (Pre), immediately after the end of the surgical procedure (T0), as well as 1 day (T1), 3 days (T3), 5 days (T5) and 7 days (T7) later. Data in bar charts are given as medians and the 95% CI. As already published throughout our workgroup, median plasma levels of the three parameters in healthy volunteers (n=30; red striped line) are presented [36]. [file 501430.f1.doc]
